# Once the rockets are up, who should care where they come down? The problem of responsibility ascription for the negative consequences of biofuel innovations

**DOI:** 10.1186/s40064-016-1758-8

**Published:** 2016-03-04

**Authors:** T. H. Tempels, H. Van den Belt

**Affiliations:** Philosophy Group, School of Social Sciences, Wageningen University, Hollandseweg 1, 6707 KN Wageningen, The Netherlands

**Keywords:** Responsible Innovation, Biofuels, Contestation, Global interconnectedness, Food-energy-environment trilemma

## Abstract

Responsible Innovation (RI) is often heralded in EU policy circles as a means to achieve ethically acceptable, sustainable innovations. Yet, conceptual questions on the specific notion of ‘responsibility’ and to what extent an innovation can be ‘responsible’ are only partly addressed. In this chapter the question of responsibility for the *indirect* negative effects of biofuel innovations is explored. While initially hailed as one of the much needed solutions in the global struggle against climate change, the use of biofuels has become increasingly criticised. It is argued that the increased production of biofuels has put smallholder farmers out of business, has given rise to increased food prices, sparking food riots in several countries, while also contributing to further environmental degradation as the demand for new biofuels requires the development of new croplands at the cost of forests and peat lands. In the current market-based system it is customary to disburden researchers and business companies from any responsibility for the more remote consequences of their actions. When harmful consequences are brought about through the mediation of (perhaps a long series of) market transactions, they are often considered inevitable and excusable and not an appropriate occasion for invoking anybody’s responsibility. But how broad is the scope of responsibility when it comes to the above mentioned social and ecological problems? By invoking the sacred duty to “innovate”, the business company could perhaps be exculpated. In our age, innovation is often so much celebrated that many negative impacts are duly accepted as the inevitable price of progress. By approaching responsibility from a perspective that takes into account the economic and ecological interconnectedness of the world, we show how the debate on Responsible Innovation in biofuels becomes tied in with global debates on economic justice and bioscarcity. In conclusion we argue that if we—assuming this interconnectedness—take the current requirements of “Responsible” Innovation seriously, it would result in a demanding practice that calls for a substantial departure from business as usual, which prompts the question to what extent it is reasonable to incorporate what are actually demands for global justice in programs for innovation.

## Background

Shortly after the turn of the new millennium, liquid biofuels for the transport sector were endorsed by official bodies in Europe and the USA as a most welcome contribution for solving the problems of energy scarcity, climate change and rural poverty, but have since become the target of so much criticism and controversy that the present situation looks like a virtual stalemate. In more recent years, research policy circles in Europe have become enamored by the idea of ‘Responsible Innovation’ (RI) or, in its more expanded version, ‘responsible research and innovation’ (RRI). This new approach for the social management of emerging technologies and innovations promises to address or avoid the sort of deep conflicts that have afflicted the trajectory of biofuels. Thus the question automatically arises if the promotion and development of biofuels might have been organized as an exercise in Responsible Innovation and whether this could have made a difference. As this is a counterfactual question (like the notorious ‘what if’ questions in historiography), it is impossible to come up with a straightforward and conclusive answer based on empirical research alone.^1^ Nonetheless, we will attempt to arrive at a plausible answer to this question by creatively combining theoretical considerations and empirical information on the initiation and implementation of biofuels policies. But first we will present a conceptual scrutiny of the twin ideas of RI and RRI.

As the expression ‘Responsible Innovation’ and its expanded version are still of recent coinage, they should properly give rise to some wonder and surprise. This new combination of words duly invites the question to what extent innovation can actually be ‘responsible’ and what sense of ‘responsibility’ might be implied in this connection. To explore this question, we start by turning to the classical authors Max Weber and John Dewey to give a provisional specification to the notion of responsibility, which can serve as a baseline for our discussion of Responsible Innovation in the area of biofuels.

For Weber as well as for Dewey responsibility means being accountable for the *foreseeable consequences* of one’s actions and implies an honest and serious attempt to actually foresee those consequences to the best of one’s ability and knowledge. Both hold that good intentions are not enough for responsible conduct. Weber famously distinguished between an ‘ethic of responsibility’ (*Verantwortungsethik*) and an ‘ethic of conviction’ (*Gesinnungsethik*). Within the former, “one has to give an account of the foreseeable results of one’s action” (or as the German original reads: “man [hat] für die (voraussehbaren) *Folgen* seines Handelns aufzukommen”), within the latter, in religious terms, “[one] does rightly and leaves the results with the Lord” (Weber 1968, p. 175). Weber unreservedly opted for the former. In a similar vein Dewey held that “our chief moral business is to become acquainted with consequences” (Dewey and Tufts 1908, p. 464). While Weber’s and Dewey’s view of responsibility as being accountable for the foreseeable consequences of one’s actions might not be spectacular—rather it seems quite a common-sense conception that is also shared by recent authors who write about the social responsibility of scientists (e.g. Douglas 2009; Forge 2008)—nonetheless, this fairly minimal and seemingly innocuous conception still proves to have real bite when used to critically examine the contemporary approaches to Responsible Innovation.

It is generally agreed that innovation is a collective process that involves many different actors and often exhibits unexpected twists and turns in its trajectory over time. The ultimate societal and environmental consequences of a particular innovation are therefore virtually unpredictable. This radical uncertainty of innovation processes, which partly springs from their collective character, is also duly recognized by advocates of Responsible Innovation: “The unpredictability of innovation is inherently linked to its collective nature” (Stilgoe et al. 2013, p. 1569; cf. Ozdemir et al. 2011). The puzzle, then, is what ‘responsibility’ could still mean under such circumstances. Following Weber and Dewey, one would be inclined to call research and innovation ‘responsible’ if and only if their protagonists are willing and able to take accountability for, or “stand up for”, the societal and environmental consequences of their endeavors (remember Weber’s phrase “aufkommen für die Folgen”). If there is practically no way to predict or foresee such consequences, however, all talk about responsibility in this context would seem to be groundless and misleading. Matters become even more complicated when we realize that innovation as a collective process partly occurs through economic transactions on the market.^2^

In the traditional liberal conception of the market system it is quite customary to relieve economic actors from any responsibility for the more remote consequences of economic competition and technological and commercial innovation. When harmful consequences are brought about cumulatively through the mediation of (perhaps a long series of) market transactions, they are often considered inevitable and excusable and not an appropriate occasion for invoking anybody’s remedial responsibility (Miller 2001, p. 458). So when one supermarket A drives supermarket B out of business “by offering a better service to customers”, we would probably be inclined to agree that in that case supermarket A bears no special responsibility to compensate supermarket B for the economic damage the latter incurred through A’s actions.

The doctrines of Corporate Social Responsibility (CSR) and Responsible Innovation (RI) run counter to this conventional wisdom. In CSR there are for instance activists in the worldwide anti-sweatshop movement, who have been quite successful in convincing Western consumers and multinational companies that they bear some responsibility for the fate of far-away workers toiling in nominally independent subcontractor firms in the initial stages of the value chain of the international garment industry (Young 2004). In a similar vein the advocates of Responsible Innovation try to mitigate the possible social and ecological problems that arise from development of novel (technological) innovations.

Nonetheless, both these currents of thought are themselves far from clear about where to draw the lines in delimiting the scope of responsibility for the social and ecological consequences of new scientific and technological developments. Moreover, widening the scope of responsibility also dramatizes the epistemic problem concerning the limited predictability of the consequences of research and innovation.

Scholars in Responsible Innovation have tried to overcome this problem of limited predictability of the consequences of innovations by turning to methods of public deliberation and public participatory decision making. The basic idea is that by involving the public in the deliberation and decision-making about an innovation project from the earliest stages on, and being “responsive” to their “concerns”, we can compensate for our limited predictive capabilities (Stilgoe et al. 2013). This way it would also be possible to evade David Collingridge’s well-known control dilemma, which holds that early on a new technology is not amenable to social control because at that stage the consequences are still unclear, while later on, when the consequences become manifest, the technology is so much entrenched that it can no longer be steered in a desirable direction (Collingridge 1980).

The trick is to use the “societal concerns” about possible future effects of an innovation *as expressed by the public in the present (real*-*time)* as a stand-in for the virtually unforeseeable future consequences and then to feed those “concerns” back into the on-going research and development work (Fisher 2005). When the innovators are genuinely responsive to the public’s concerns, they should be able to curb these possible future negative effects.

Various forms of foresight and forecasting figure prominently in the armamentarium of Responsible Innovation—indeed, ‘anticipation’ is considered one of its central dimensions, next to ‘reflexivity’, ‘inclusion’ and ‘responsiveness’ (Stilgoe et al. 2013)—but the meaning of these words seems to deviate from their normal sense. As David Guston clarifies, “anticipatory governance involves a rejection of prediction but an embrace of an approach to foresight we call anticipation, which casts multiple, plausible futures as objects for deliberation rather than a single predicted future as an object of pursuit” (Guston 2010, p. 434). What is fed as an input into public deliberation and decision-making about technology development is thus not a forecast of the future, but a menu of visions of various possible futures, which can then be taken into account in the processes of research and innovation.

### The turn to public engagement

One can wonder, however, why such visions have to be taken seriously as a basis for concern calling for adjustments in the innovation trajectory if they are only slightly plausible. The shift from predicting effects to being responsive to societal concerns also explains why public participation or public engagement is considered an essential part of Responsible Innovation. A central role for the public is also prescribed by the ideal of a deliberative democracy, as set out, for instance, in Habermas’s discourse ethics. In a situation of genuinely power-free communication, according to Habermas, the only force that counts is the force of the better argument. Following this line of ethical reasoning, Annelies Balkema and Auke Pols argue that the responsibilities of policymakers in Responsible Innovation “include making sure that actual stakeholders are identified as such and are invited into the discussion, stakeholders representing all relevant views can participate in the discussion, and that power differences between stakeholders are compensated for as much as possible, in order to create a level playing field” (Balkema and Pols 2015, p. 8). Moreover, the participants in the discussion have to engage in rational argumentation. They are expected to give “all values and arguments due consideration, and not suppressing or excluding any relevant argument” (ibid.).

Critics, however, claim that discourse ethics is unduly idealistic and that the procedural requirements for communicative rationality are far too demanding to be practically implemented in real-life institutional settings. Just to call for an ‘open’ debate is a meaningless gesture if no “frame” is provided to define the issues at stake—without a frame, there is no disagreement but only indifference. Setting a frame, however, implies establishing relationships of power (Torgersen and Schmidt 2013). Some critics go further and call into question not just the practical feasibility but the normative desirability of the ideal of power-free communication. The Danish economic geographer Bent Flyvbjerg, for one, considers the very attempt to purify communicative rationality from power, rhetoric and strategic opportunism as fundamentally misguided (Flyvbjerg 1998), while the Belgian philosopher Chantal Mouffe insists that democratic politics is not a utopian quest for an unforced consensus, but a pluralistic process of contestation and confrontation marked by ineradicable antagonism (Mouffe 2009).

These alternative views in political philosophy also lead to a more skeptical assessment of the role and potential of public engagement in Responsible Innovation. Its advocates often lament the *strategic* reason policymakers sometimes invoke for setting up participation initiatives as a way to ensure public support for the delivery of a pre-committed policy (Owen et al. 2012, p. 753; Stilgoe et al. 2013, p. 1573). However, it would be amazing if such strategic motives were absent given what is at stake for the various parties. After all, in the battle for the hearts and minds of the populace to which a public participation exercise ultimately boils down, each interested party has its own axe to grind (cf. Van Oudheusden 2014). Furthermore, deliberative initiatives do not necessarily guarantee full societal consensus, as groups with radical or non-reformist worldviews tend to be excluded, or exclude themselves, from participating in the deliberation (Schouten et al. 2012). A case in point is the almost continuous public engagement with agricultural biotechnology: “From surveys to focus groups to citizen juries, GM crops have probably been engaged with more than any other technology, but this has not helped to build societal consensus in Europe” (Tait and Barker 2011, p. 766). These theoretical insights and practical findings give reason to at least tone down the high expectations that discourse ethics and deliberative democracy have vested in public participation initiatives.

From a business angle, the emphasis on reflexivity and “opening up” rather than “closing down” (Stirling 2008) in current versions of Responsible Innovation may also be problematic. Understandably, companies will be reluctant to commit large funds to risky innovative projects as long as everything is up for grabs. Critical social scientists have perfected the art of questioning framing assumptions, but have largely ignored the difficult task of bringing a public debate to a timely conclusion.

In many European countries public engagement with new technologies (e.g. nanotechnology) is routinely organized as a part of official research and innovation policy, but laypersons are often reluctant to participate. The professional organizers of participatory events have sometimes much difficulty to recruit sufficient numbers of willing participants. On occasion, citizens’ dialogue meetings take place—without citizens (Bogner 2012, p. 509). One avowed reason why ordinary citizens show little enthusiasm to take part in “upstream engagement” is that at an early stage of development the possible future impacts of a new technology are still unclear even to the experts. Perhaps the attempt to square the circle of limited predictive capacity by involving the wider public is bound to fail after all.

### Provisional balance-sheet

Our preliminary, theoretically informed analysis of the notion of Responsible Innovation given above thus already raises several skeptical points. The overall impression is that the currently popular approach would still fall short of adequately addressing the problem of “responsibility” in the context of research, innovation and technology development. According to Weber and Dewey, one is to be held responsible for the “foreseeable” consequences of one’s actions, but in the context of RI the problem is precisely the limited “foreseeability” of the future consequences of innovation, despite the fact that RI virtually amounts to a sustained attempt at exercising “foresight”. Nor can this problem, it seems, be solved or compensated for by engaging the wider public. It remains to be seen to what extent the problematic issues that we identified above actually emerge in concrete processes of research and innovation in novel technologies and how they are (or could be) dealt with in such settings. We thus turn in the next section to the global biofuel debate in order to examine an actual example. We first set out how the process of biofuel innovation and the corresponding biofuel policies have developed over the past years and then explore the negative direct and especially indirect effects which have been contestably attributed to this once almost unilaterally welcomed solution in the global quest for sustainable energy.

## A thumbnail sketch of US and EU biofuels policy

At the start of the new millennium liquid biofuels for the transport sector were hailed both in the USA and in Europe as a welcome climate-neutral solution for the problem of energy supply. By mandatory blending of bioethanol and biodiesel obtained from agricultural crops with petrol and diesel, a modest initial impetus could be given for a necessary transition towards a future ‘bio-economy’, which would no longer be based on fossil resources. In due course these so-called ‘first-generation’ biofuels would be followed by more advanced (‘second-generation’ and even ‘third-generation’) biofuels.^3^

However, the honeymoon period for the first generation of biofuels was not to last for long (Van den Belt et al. 2008, pp. 37–72). Their reputation received a severe blow in the year 2007 when it appeared that the competition of the increasing production of biofuels with food production (“fuels versus foods”) was one of the underlying causes of the rise in food prices that led to food riots among the urban poor in several developing countries. The following year the journal *Science* published two quantitative studies (Fargione et al. 2008; Searchinger et al. 2008), which suggested that the large-scale cultivation of biofuel crops might on balance *increase* rather than *decrease* greenhouse gas (GHG) emissions by inducing (direct and indirect) changes in land use. Environmental NGOs that had initially supported the switch to biofuels, now turned against it. Developmental NGOs joined the opposition on the grounds that a boom in biofuels would endanger food security and lead to land grabbing and the further marginalization of vulnerable groups in developing countries. Proponents of biofuels (encompassing biotech companies, farmers and researchers) often admitted that the first generation of biofuels is far from perfect, but pinned their hopes on the development of more advanced generations, which would fulfill the promise of climate neutrality after all and mitigate or even eliminate any competition with food production. In their view the first generation, however problematic, is an indispensable stepping stone towards later generations and cannot be skipped.

In recent years much of the debate about biofuels has concentrated on the details of the regulatory regime aimed at stimulating and channeling the deployment and development of these alternatives for fossil transport fuels. Both the US government and the EU rely on mandatory blending targets as their primary policy instrument. Thus, the European Commission Renewable Energy Directive of 2009 envisages that 10 % of all transport fuel must come from renewable sources (mainly biofuels) by 2020. The EU also sets out some minimal sustainability criteria. Biofuels must achieve greenhouse gas savings of at least 35 % in comparison to fossil fuels, cannot be grown in areas converted from land with previously high carbon stock such as wetlands and forests, and cannot be obtained from high biodiversity areas like primary forests. In 2012 the European Commission (EC) issued a proposal to amend both Directives with the aim of limiting the use of first-generation biofuels to only half of the 10 % target for 2020 and to stimulate a more rapid development and deployment of second-generation and third-generation biofuels (EC 2012).

The Commission touched upon a very sensitive issue when it also suggested in 2012 that providers and EU member countries should *report* on emissions that might be caused by “indirect land use change” (ILUC).^4^ The issue of ILUC is a bone of contention among the various parties, meeting all the criteria of a “wicked problem” (Palmer 2012). While environmental and developmental NGOs insist on the need to actually include (and not just report about) ILUC estimations when determining which fuels may count as renewable, industry representatives denounce their use because they claim there is no proper scientific methodology on which they might be based. At the time of writing, the ILUC issue and the problem of how to promote the development of second-generation biofuels, are still unresolved within the European institutions.

## The indirect social and environmental effects of biofuels: a contested issue

When it comes to the indirect social and environmental effects of the production and use of biofuels we see an ongoing discussion on both whether there are such problems and which actor(s) should carry responsibility for these issues.

ILUC effects have been controversial ever since the quantitative study by Tim Searchinger et al. was published in 2008. Searchinger and his colleagues concentrated on the indirect effects of expanding the area of biofuel crops. They calculated that a planned increase of US corn ethanol production of 56 billion liters in 2016, diverting 12.8 million ha of US cropland from food and feed production and inducing the cultivation of an extra 10.8 million ha of land elsewhere and on other continents, would nearly double GHG emissions over 30 years and increase greenhouse gases for 167 years. They thus conclude: “Use of good cropland to expand biofuels will probably exacerbate global warming in a manner similar to directly converting forest and grasslands” (Searchinger et al. 2008, p. 1240).

Not all actors in the biofuel debate have been keen to accept the findings of the Searchinger report. In Europe representatives of the biofuel industry have been complaining about the unfavorable investment climate resulting from the current political indecision in Europe, resulting from the research on ILUC effects. For instance, Robert Wright, secretary-general of *ePure*, the association of the European ethanol industry, laments that the “ILUC cloud” hanging over the industry and the Commission’s policy turn have destroyed investor confidence: “For investments to take place we need stable, clear and long-term policies and no policy u-turns” (Wright quoted in Maler 2014). In a similar vein the Danish biotech firm Novozymes, which has a strong interest in the development of second-generation biofuels (because it specializes in making enzymes, which will be needed if cellulose is to be broken down), opposes accounting for ILUC effects. As a company spokesman explains: “ILUC is a controversial concept. The belief behind the concept is that the displacement of other land-using activities, caused by biofuels expansion, contributes to greenhouse gas emissions elsewhere—and this should be factored into the lifecycle analysis of biofuels. As such, ILUC cannot be calculated but only modelled, and variations in models show very different figures. It is therefore also very difficult to base legislation on it” (Amrani 2015; Novozymes, n.d.).

In the United States the findings of the Searchinger study have been severely criticized by the Biotechnology Industry Organization (BIO) and the New Fuels Alliance, industry groups with an important stake in US biofuels production. They believed that Searchinger and his team underestimated the possibilities of technological progress in biofuels and agriculture generally and they also held that the inclusion of indirect effects like land use changes would necessitate the use of “very complex econometric models” (BIO 2008). The two industry groups suggested to cling to Standard Life Cycle Analyses (which exclude consideration of indirect effects) on the grounds that we do not have the sophisticated models to adequately deal with land-use changes (BIO 2008; New Fuels Alliance 2008). It can be readily admitted that adequately modeling land-use changes is extremely difficult, but the industry response is still remarkable. Their position actually amounts to turning a blind eye to such indirect effects, perhaps in the hope that they will not be there if we do not look for them.

Not surprisingly, the US biofuels industry declined any responsibility for increased GHG emissions through land-use changes. They also tried to lay this responsibility at the doors of governments, as the Biotechnology Industry Organization declared: “Indirect land use changes are a function of land use policy. Sustainable biofuels production must go hand-in-hand with sustainable land use policy” (BIO 2008). In other words, it is up to the governments of biofuel producing countries like Brazil, Indonesia or Malaysia to prevent that the pressure on land to meet the increased demand for biofuel will lead to further deforestation.

What the organization did not say is that such a policy, if it were in place, might reduce food production. As the Searchinger study observes: “Effective controls on land conversion would constrain the major source of new supply to meet increased biofuel demands, resulting in less additional cropland and higher market prices as markets seek equilibrium. *In that event, more greenhouse benefits would stem in reality from reduced food consumption*” (Searchinger et al. 2008, p. 1240; our italics).^5^ It seems that using land for the growing of biofuel crops competes with using land for food production or keeping it as forest or wilderness areas. There is in fact a “food-energy-environment trilemma” (Tilman et al. 2009). This trilemma can be evaded by biofuels from waste streams and from perennials on degraded farmland or from algae grown in the desert (there is not much carbon in the desert). The scope for such possibilities appears however rather limited (cf. Hansen 2014).

Land conversion due to the worldwide boom in biofuels may also have social consequences, e.g. in the form of indigenous peoples being evicted from their traditional homelands to make room for palm oil plantations or farmland for growing biofuel crops. In 2012 alone the organization *Sawit Watch* (Palm Oil Watch) reported 660 land conflicts raging over biofuels cultivation in Indonesia (EurActive 2012).

Who is to be held accountable for such consequences? In 2008 the European Parliament made a first attempt to take responsibility for these negative consequences as it proposed to include social aspects like land rights of local communities and fair remuneration of workers in the sustainability criteria for biofuels. However, it later decided against mandatory social criteria “because of the difficulty to verify the link between individual biofuel consignments and the respect of these particular criteria” (Levidow 2013, p. 217; quoting from a Commission document). An additional reason was the fear that mandatory certification for social standards, as a potential violation of global trade rules, could face challenges by producer countries before the WTO dispute settlement mechanism (*ibid.*). The upshot is that the responsibility for protecting indigenous land rights and workers’ rights, which may come under pressure as an indirect consequence of the increased European demand for biofuels, is not accepted by the European Union, but is put into the hands of the governments of the producer countries. From a legal and political angle, it is arguable that this is precisely where such responsibility properly belongs. However, in practice many governments in developing countries are not actively defending the customary land tenure rights or labor rights of vulnerable groups in society, but are more or less complicit in what has unofficially been termed ‘land grabbing’ to such an extent that a new massive global wave of ‘accumulation by dispossession’ is going on (White and Dasgupta 2010).

All this leaves us with an interesting but difficult philosophical puzzle about the proper distribution of responsibilities. When political institutions take the lead in designing a regulatory regime for the development and deployment of biofuels, it is natural to also grant them primary responsibility for the social and environmental consequences. However, this does not remove the responsibility of other parties like business companies, industry associations and civil society organizations, if only because they all take policy stances and are actively engaged in trying to influence the regulatory regime through lobbying (Miller 2001; Young 2011).

The setting of ambitious blending targets for the use of biofuels in the US and the EU sets in motion a long train of causation, which arguably ends up with the further marginalization of vulnerable social groups in developing countries, the clearance of forests and peat lands and the conversion of grasslands into croplands, the loss of biodiversity and perhaps a reduction of food production and perhaps also a net increase in GHG emissions. However, this is not a train of (purely) *physical* causation, but (at least partly) of *social* causation, meaning that it occurs through the economic and political *decisions* of all the actors that make up the successive links of the chain. It would seem, therefore, that the US and European governments operating at the start of the chain could legitimately pass the responsibility for averting certain negative social and environmental consequences on to later links in the chain, to wit, to the governments in the developing countries where these consequences are bound to manifest themselves; after all, it is in the power of these latter governments to prevent these effects from happening by properly protecting nature and the rights of the population. But what if the governments in the developing countries refuse or fail to take up this responsibility? Does (part of) it then devolve back to the US and the EU governments? Could these governments justifiably have decided to pass on the responsibility to their Third-World counterparts in the first place on the highly plausible assumption that the latter would not take it up? After all, one important tenet of Weber’s *Verantwortungsethik* is that we take the world as it is, not as it ideally should be. And should we only talk about the responsibilities of political agencies in this context? What about the responsibilities of business companies, scientists, and civil society organizations?

In this connection it is interesting to note that the public authorities of the EU did not themselves take steps to ensure compliance with the (minimal) sustainability criteria the Renewable Energy Directive (RED) sets for biofuels, but actually relied on stakeholder platforms consisting of business organizations and NGOs to implement voluntary schemes for certifying “sustainable” biofuels (Richardson 2014). Thus the Roundtable on Sustainable Palm Oil (RSPO) created in 2010 a voluntary add-on to the existing RSPO standard to enable producers to comply with the EU’s sustainability criteria (RSPO-RED). Similar schemes have been set up by other stakeholder platforms like Bonsucra (sugarcane-based biofuels from Brazil) or the Roundtable for Sustainable Biofuels. In 2012, the European Commission recognized several of these voluntary certification schemes. It can be argued that the public authorities in the EU have passed the practical responsibility for ensuring compliance with their sustainability criteria onto the private sector. Although ‘reformist’ NGOs like the Worldwide Fund for Nature are generally willing to participate in such stakeholder platforms, more radical environmental organizations like Friends of the Earth tend to keep aloof from them (Schouten et al. 2012). Radical NGOs point out that, by their very nature, certification schemes cannot deal with ILUC effects. Even if the certification standard went beyond the quite minimal EU criteria, it would still be unable to capture such effects (Richardson 2014).

The above discussion suggests that current views on Responsible Innovation might not offer us much guidance on how to set up an intelligent policy for biofuels. It seems that societal decision-making has got stuck in a stalemate where different actors favor conflicting policies and mutually block each other’s preferred options. The biofuels case amply illustrates the limited predictability of the social and environmental impacts of innovations, but does not bear out the expectation that being “responsive” to the expressed “concerns” of the public and civil society can somehow make up for this deficiency. Disagreement immediately resurfaces about which concerns are serious enough to be addressed and what constitutes an adequate and sufficient response. Policy-makers have indeed attempted to address concerns about biodiversity loss and enhanced greenhouse gas emissions by setting additional sustainability criteria, but these measures are themselves contested and have often been judged insufficient. Most revealing is the endless dispute about ILUC effects. The EU has commissioned no less than fifteen scientific studies on this topic, all with different and highly divergent outcomes (EurActiv 2015).

In order to get a clearer understanding of what responsible action could mean within the biofuel debate, we will further reflect on the concept of responsibility and connect this to Responsible Innovation in the biofuel debate.

## Reframing the ethical issues: responsibility in an interconnected world

In this section we will reflect further on the deeper roots of the difficulty of “responsible” innovation in the biofuels field. Besides the widely recognized uncertainty of innovation processes in general and the limited predictability of their effects, we think we should also point to the terrestrial reach of biofuel innovations, which are played out, so to say, in the global theatre of an *economically and ecologically interconnected* world. We will build on an article written by the American bioethicist Paul Thompson to elaborate on the economic aspect of this interconnected world and on an article by the Danish sociologist Janus Hansen to elaborate on the ecological aspect. These two aspects will then have to be combined. Proponents of biofuels generally hold that the dilemma of “food versus fuel” which still afflicts the first generation of biofuels will be overcome when the progress of technology allows us to switch to the more advanced second en third generations in the near and foreseeable future. Even the more comprehensive “food-energy-environment trilemma” will then likely be overcome thanks to gains in efficiency. Both Thompson and Hansen, however, offer arguments to the effect that the expected technological progress is actually unable to resolve the underlying ethical dilemmas.

### Thompson’s economic interconnectedness

Thompson argues that the focus on non-food crops to escape from the food-versus-fuel dilemma misses the mark. If a farmer grew miscanthus or jatropha instead of maize as a fuel crop, this would just as well affect the supply of maize for food, albeit more indirectly. Ultimately, due to the interconnection of the markets for agricultural products, all the various forms of land use are in competition with each other. Nor would the development of cellulosic ethanol resolve the tension. As Thompson remarks, “the ethical issue is *not* one of finding technically feasible alternatives to crops currently used for food. It is rather in the socio-economic priorities that dictate which of many technically feasible possibilities will be realized in practice” (Thompson 2012, p. 347). In fact, the successful development of cellulosic ethanol would even put food and fuel production into more *direct* competition with each other, at least theoretically, because if cellulose can be converted into sugar and starch the latter could theoretically also be processed further into food rather than being transformed into ethanol for blending with petrol. The fact that no practical technologist would pursue this ‘theoretical’ goal only reflects the relative value judgments that are embodied in current world prices for food and fuel and thus underscores precisely the ethical point Thompson is trying to make. As a society we place so much more value on driving our cars than on ending world hunger, that we uncritically accept the relative prices of fuel and food that express our valuations as the correct signals for economic action. This not only holds for allocating given resources in the market, but also for decisions to pursue certain research lines aimed at developing new resources. Here we find a similar moral issue: “The fact that poor people cannot compete with rich people for basic grains is structurally isomorphic to the fact that they cannot compete with the rich when it comes to land use allocation or to capital investment in new technology” (*ibid*., p. 349). In line with Thompson’s analysis we can reframe and generalize the underlying ethical issue in the food-versus-fuel dilemma. The ultimate ethical problem, then, is that the priorities and the claims of the rich carry so much more weight in world markets than the needs of the poor, both in determining what gets produced and in determining the direction of the research effort. Just as pharmaceutical research is skewed in serving the desires of the rich rather than the needs of the poor (by developing drugs against baldness or erectile dysfunction rather than against Chagas disease), so the biotechnological research aiming at advanced generations of biofuels is similarly driven first and foremost by the priorities of the rich (Pogge 2005). In a finite world this will lead to a further displacement of the needs of the poor. We may add to Thompson’s analysis that it is mainly the institutional mechanism of the patent system (itself a global system since the TRIPS Agreement of 1994) that connects the research effort to the wants and priorities of the rich (Timmermann and Van den Belt 2013; Pogge 2005).

### Hansen’s ecological interconnectedness

One of the basic tenets of ecology is that we live in a world of finite resources. This view is orthogonal to the optimistic faith in progress, according to which our knowledge grows at an exponential rate and the potentialities of science and technology are virtually without limit—so much so that they can ultimately also overcome the finiteness of earthly resources. These two views have been pitted against each other ever since the classical dispute between the marquis de Condorcet and the Reverend Thomas Malthus at the end of the 18th century. Janus Hansen (2014) discerns in the Danish debate on biofuels two distinct scientific perspectives which can be seen as modern versions of these classical positions. With the technologically optimistic position corresponds what Hansen calls the “reductionist biorefinery perspective”; while the more pessimistic position which stresses the limits to growth corresponds to what he calls the “holistic bioscarcity perspective” (Hansen 2014). Both perspectives derive from different scientific fields. The first perspective originates from biochemistry and neighboring disciplines (including molecular biology, biotechnology and synthetic biology), the second from life-cycle analysis, environmental science and ecology. The first works upward from the molecular level while the second works downward from the global resource situation. The two perspectives form the scientific core of two different *advocacy coalitions*, which act in the political arena to lobby for their preferred policy lines. Thus the reductionist biorefinery perspective is linked to the coalition of *biofuels optimists* consisting of the enzyme-producing biotech companies Novozymes and Danisco, biofuels producers (e.g. DONG) and agricultural interests. The holistic bioscarcity perspective is linked to the coalition of *biofuels skeptics*, comprising environmental and developmental NGOs but also parts of the conventional energy sector.

We believe the distinction made by Hansen between these two perspectives serves not only to clarify the Danish debate on biofuels, but also sheds light on similar debates elsewhere. The optimistic perspective is informed by the guiding vision of the *biorefinery* as “a generic process technology, whereby any biomass can be transformed into a plant-based equivalent of crude oil” (*ibid*., 82). In this perspective, biomass is seen as an abundant resource. It is up to our scientific ingenuity to fully exploit the potential of this resource (e.g. by enzymatic degradation of celluloses). Any technical problems confronting us in this trajectory are just challenges that can be overcome by (further) research and innovation, provided the right regulatory policies (like blending targets, subsidies and tax credits) are in place to provide adequate economic incentives. While the adherents of this perspective admit that first-generation biofuels are somewhat problematic, they also hold that they are a necessary first step to develop an infrastructure for the second and third generation. Anyway, with the arrival of these more advanced generations the food-versus-fuel dilemma will be finally solved.

In the pessimist perspective, by contrast, biomass is considered a scarce resource. The biofuels skeptics are less willing to mortgage the future by signing up today on the seductive promises of our scientific ingenuity and hold that the distinction between the first generation and more advanced generations of biofuels ultimately does not make much of a difference. They notice that Western countries like Denmark, despite all talk about biomass as an abundant resource, are unable to supply their own domestic needs and rely on large-scale imports of biomass from developing countries. They fear that the expected growth in biomass consumption, not just due to increased use of liquid biofuels for road transport, but also to satisfy other virtually insatiable Western desires and priorities, will negate any sustainability gains and shift the burden onto fragile ecologies and communities. Or as Hansen remarks, “skeptics … see biofuels as a neo-colonial project, which will inevitably work to the disadvantage of vulnerable people and ecosystems” (ibid., 92).

### Acting in an economically and ecologically interconnected world

As an analyst Hansen takes a neutral stance towards the two perspectives. We think, however, that the “holistic bioscarcity perspective” should at least be taken seriously as a plausible approach that tries to do justice to the economic and ecological interconnectedness of our global world.^6^ In the economy of nature, just as in our human economy, there is no such thing as a free lunch. To us it seems that the ecological heel of Achilles for biofuels is precisely the incredible amounts of biomass that must be fed to this hungry monster. Even the latest representatives of scientific optimism, the synthetic biologists working on advanced biofuels, are confronted with this problem. As an environmental NGO writes in a critical report, “Synthetic biology enthusiasts work under the false assumption that there will be an endless supply of biomass and land to fuel their biofuels revolution” (Friends of the Earth 2010, 15).

This criticism is supported by the estimation of the maximally available worldwide potential of primary bio-energy, which was made in 2012 by Kolby Smith and his colleagues on the basis of satellite data and which placed an upper limit to the possible contribution of biofuels. They concluded that this potential would be between 35 and 108 % of worldwide primary energy consumption in the year 2009 (Kolby Smith et al. 2012). Because only one-third of this potential is realistically available, the newly estimated upper limit is about four times less than earlier projections. Thus an economy based entirely on biofuels would not even come close to meeting the needs of an energy-hungry world: 5 to 15 percent of the projected energy demand for 2050 at most (Kolby Smith et al. 2012, p. 921).

Proponents of biofuels point at the availability of all kinds of agricultural waste and of marginal lands that could be deployed for the production of the required biomass. They seem to count liberally on the availability of unused or underused resources, and according to their opponents, they tend to do the accounting in their own favor, because in the process they ignore the fact that so-called “waste” is actually being used for various useful purposes and that “marginal” lands provide a source of livelihood to smallholders, nomadic herdsmen or indigenous peoples (Friends of the Earth 2010, p. 14). Anthropologist Jennifer Baka has shown through detailed fieldwork in South India that the so-called “wastelands” designated by the government for the cultivation of the biofuel crop jathropha had actually been much more productive in their previous capacity, both in terms of bioenergy production and other useful applications; the main net result of the whole process was a massive “land grabbing” (Baka 2013, 2014). When so-called marginal lands are taken into exploitation, there is also a potential conflict with nature conservation, creating an additional opposition, namely one of flora-versus-fuel next to the food-versus-fuel dilemma (The Economist 2013).

In the socio-economic constellation of our contemporary world, the development of biofuels will most probably result in indirect external effects like the displacement of indigenous communities from marginal lands and the replacement of natural ecosystems by biomass monocultures. The research effort, induced by the incentive of patents, is one-sidedly oriented to aims that promise a tangible profit. In the process, other aims—the ones that are not supported by an effective demand backed by purchasing power—will be easily sacrificed. These wider connections were also broached with admirable clarity in a debate between conservationists and synthetic biologists:

“Synthetic life delivers private benefits. Many forms of life being developed by synthetic biology are being patented. The benefits provided by these organisms will reflect the economic interests of those able to invest in and develop them. This may well favor applications in existing industrial processes and commodity chains (energy, agriculture, aquaculture) and the operations of large business corporations. *Impacts on the wider environment will tend to be treated as an externality*. Knock-on impacts of price and other economic changes on smaller producers (e.g. smallholder farmers) will affect their decisions about land conversion and management, and hence future patterns of biodiversity loss. How will a balance be struck between private risk and gain versus public benefit and safety?” (Redford et al. 2013; our italics).

Are researchers and companies co-responsible for such indirect effects or can they wash their hands in innocence? A typical response to this question was given by Jay Keasling, the Berkeley professor of synthetic biology who developed with his team a synthetic precursor of artemisinin, a valuable drug against malaria. This achievement is often presented as the poster child of synthetic biology, but there is also a darker side to it. Making the artemisinin precursor in a fermentation vat with genetically engineered yeasts will cause tens of thousands of Asian and African farmers who formerly extracted the natural artemisinin product from their sweet wormwood crop (*Artemisia annua*) to lose their livelihoods. When confronted with this consequence of his scientific work, Keasling replied:

“I don’t make the decision about what gets produced […] The marketplace decides. What I do is provide more options” (Keasling quoted in Callaway 2013, p. 281).

This particular answer led one uncharitable critic to refer to the famous lines from Tom Lehrer’s song on the German rocket scientist Wernher von Braun:

“Once the rockets are up, who cares where they come down.

That’s not my department, says Wernher von Braun” (quoted in Shanks 2013).

Given the earlier discussion on economic and ecological interconnectedness it would seem that Keasling cannot hide in good faith behind the invisible hand of an anonymous market. The extra options he provides as a synthetic biologist have been deliberately chosen rather than randomly selected.

What the above discussion makes clear is that there are sufficient reasons to attribute not just responsibility to governmental actors, but that companies and researchers are connected as well to the negative indirect effects of the biofuel innovations, which gives them at least a certain degree of co-responsibility.

## Conclusion

The example of biofuels shows that there are limits to the extent to which both political bodies and corporate actors can effectively assume responsibility for the likely consequences of innovations and novel technologies within the current global economy. This is not to deny that government institutions and companies did indeed take *some* responsibility for the consequences of the development of biofuels. In accordance with the precepts of Responsible Innovation, the EU showed itself at least to some extent “responsive” to the concerns expressed by environmental NGOs, e.g. by adjusting policy targets or issuing additional sustainability requirements. Companies, on their part, followed voluntary schemes for certifying sustainable biofuels. However, whether these responses are fully adequate and sufficient is still the subject of intense debate.

The preceding analysis highlights that there was permanent contestation about the exact consequences of the use of biofuels and no definitive scientific consensus crystallized about the *attribution* of alleged consequences to the actions and measures that could be held (causally) responsible for them. That is not surprising, because we are dealing here with a ‘wicked’ problem, as the long chain from initial cause to ultimate effect includes an endless number of links involving many market transactions and complex natural mechanisms. This opens the possibility for political and corporate actors to wash their hands in innocence, or rather in ignorance; with some right they may hide themselves behind a veil of ignorance. It is always difficult to tell where legitimate ignorance passes into wilful ignorance. Connectedness is thus on the one hand a general ground for assigning responsibility to several multiple actors, as in Young’s theoretical approach (cf. Young 2011), but on the other hand it tends to assume such unwieldy dimensions here that the ‘wicked’ consequences of technological innovation in our economically and ecologically highly interconnected world seem to escape the practical grasp of any responsibilities that can reasonably be assigned to eligible actors, short of changing the basic rules of the current global system.

Another way to express the same insight is to say that the procedural requirements of inclusion, foresight, reflexivity and responsiveness that are held to characterize Responsible Innovation, *if taken really seriously*, would be extremely demanding. They could for instance require continuous responsiveness to newly arising (in-)direct negative effects of innovations. It would no longer be acceptable to treat negative societal and environmental effects as externalities that are passed on through market transactions and ecological chain reactions, so that unknown people living somewhere else—usually the most marginalized and vulnerable—must ultimately bear the brunt of the inevitable fallout of our attempts to improve the quality of our lives. Ultimately, it would seem to call for a radical departure from business as usual and one might even argue that this would actually presume the existence of a world that is much more equitable and just than our present one.

The obvious question then arises as to whether the criteria for Responsible Innovation should be used across the board for all innovation processes or only applied selectively to particular projects of technology development. In the latter, more likely case, the innovators who have been singled out for special attention might justifiably complain about double standards: why are they the only ones for whom the bar of responsibility has been raised to such an incredibly high level? If the criteria would be applied across the board, innovators might still complain about discrimination of emerging technologies as against already established technologies. One is reminded of venture capitalist and biofuels enthusiast Vinod Khosla, who in 2007 entered the food-versus-fuel debate by claiming that using maize to produce a gallon of bio-ethanol was much more useful to society than using the same amount of the crop for producing a kilogram of beef on your table. Why was all the social criticism so unfairly addressed to biofuels?

If we conceptualize responsibility within Thompson’s and Hansen’s frameworks of interconnectedness, it would seem that Responsible Innovation, if its ambitions are taken seriously, has little chance to survive in the current context of research and innovation policies that are primarily oriented to improving the competitiveness of European business companies on the world market. The former EU Commissioner for research, innovation and science who officially launched the idea of Responsible Research and Innovation, Máire Georghegan-Quinn, also declared in the European Parliament: “We are looking to address all the bottlenecks in innovation. We want to translate our excellent research into products that our companies can bring to market. Europe needs to remove barriers that get in the way of getting from research to retail” (EurActiv 2010). But, how responsible can one truly be if you have to move as quickly as possible “from research to retail”?

## Footnotes

1 We like to thank the anonymous reviewer who pointed out the counterfactual character of our central question, and the possible difficulties this brings along.

2 According to Schumpeter, such transactions are in fact a defining characteristic of ‘innovation’; without them, there is only ‘invention’ but no real ‘innovation’(Schumpeter 1939, p. 84).

3 “First-generation biofuels are produced from crops which are also used for food production or animal feedstuff. Second-generation biofuels are produced from biomass which cannot serve as food or feedstuff […]. Proponents claim that second-generation fuels will remove competition with food production. Some observers also talk about third-generation fuels derived from algae, which is claimed to alter energy production completely in the future. Whereas the first-generation fuels are already in wide commercial circulation, second-generation ones are still not commercially viable and third-generation fuels are still in early R&D stages” (Hansen 2014, p. 80).

4 “Decisions regarding land for biofuels can have adverse consequences far beyond the land directly in question. For example, if fertile land now used for food crops (such as corn, soybeans, palm nuts, or rapeseed) is used to produce bioenergy, this could lead, elsewhere in the world, to farmers clearing wild lands to meet displaced demand for crops. In this way, indirect land-use effects of biofuels can lead to extra greenhouse gas emissions, biodiversity loss, and higher food prices” (Tilman et al. 2009, p. 271).

5 In a more recent article, Searchinger et al. (2015) show that according to the three major models that inform US and EU biofuels policy, ethanol policies in fact are relying on decreases in food consumption to generate savings in greenhouse gas emissions.

6 By taking this stance, our position is tilting towards that of the “biofuels skeptics” as we adopt a more critical attitude towards current biofuel policies. We recognize that this position does not fully take into account the unresolved epistemic uncertainty policy makers are still confronted with (the expectations of the biofuels optimists might turn out to be right, after all). Therefore our refusal to take out a mortgage on the future by counting on robust technological progress may entail some unfairness in our assessment, however we defend this position on ethical and prudential grounds.
